# Invasive Salmonellosis among Children Admitted to a Rural Tanzanian Hospital and a Comparison with Previous Studies

**DOI:** 10.1371/journal.pone.0009244

**Published:** 2010-02-16

**Authors:** George Mtove, Ben Amos, Lorenz von Seidlein, Ilse Hendriksen, Abraham Mwambuli, Juma Kimera, Rajabu Mallahiyo, Deok Ryun Kim, R. Leon Ochiai, John D. Clemens, Hugh Reyburn, Stephen Magesa, Jacqueline L. Deen

**Affiliations:** 1 National Institute for Medical Research - Amani Centre, Tanga, Tanzania; 2 Joint Malaria Programme, Moshi, Tanzania; 3 Teule Hospital, Muheza, Tanga, Tanzania; 4 International Vaccine Institute, Seoul, Korea; 5 Mahidol Oxford Research Unit, Bangkok, Thailand; 6 London School of Hygiene and Tropical Medicine, London, United Kingdom; Sabin Vaccine Institute, United States of America

## Abstract

**Background:**

The importance of invasive salmonellosis in African children is well recognized but there is inadequate information on these infections. We conducted a fever surveillance study in a Tanzanian rural hospital to estimate the case fraction of invasive salmonellosis among pediatric admissions, examine associations with common co-morbidities and describe its clinical features. We compared our main findings with those from previous studies among children in sub-Saharan Africa.

**Methodology/Principal Findings:**

From 1 March 2008 to 28 Feb 2009, 1,502 children were enrolled into the study. We collected clinical information and blood for point of care tests, culture, and diagnosis of malaria and HIV. We analyzed the clinical features on admission and outcome by laboratory-confirmed diagnosis. Pathogenic bacteria were isolated from the blood of 156 (10%) children, of which 14 (9%) were *S*. *typhi*, 45 (29%) were NTS and 97 (62%) were other pathogenic bacteria. Invasive salmonellosis accounted for 59/156 (38%) bacteremic children. Children with typhoid fever were significantly older and presented with a longer duration of fever. NTS infections were significantly associated with prior antimalarial treatment, malarial complications and with a high risk for death.

**Conclusions/Significance:**

Invasive salmonellosis, particularly NTS infection, is an important cause of febrile disease among hospitalized children in our rural Tanzanian setting. Previous studies showed considerable variation in the case fraction of *S. typhi* and NTS infections. Certain suggestive clinical features (such as older age and long duration of fever for typhoid whereas concomitant malaria, anemia, jaundice and hypoglycemia for NTS infection) may be used to distinguish invasive salmonellosis from other severe febrile illness.

## Introduction

*Salmonella enterica* serotype Typhi (*S*. *typhi*) and several non-Typhi serotypes of *S. Enterica* (NTS) are important causes of childhood bacteremia in African children [1]. Although the data is sparse, there seems to be a relatively low burden of pediatric typhoid fever across sub-Saharan Africa [2] whereas NTS has consistently been reported as a leading cause of bacteremia in African children [3], [4]. In contrast to industrialized countries where NTS usually causes a self-limited gastroenteritis, invasive disease frequently occurs in sub-Saharan Africa with high case fatality rates among hospitalized children [1], [3], [4].

There is inadequate information on invasive salmonellosis in sub-Saharan Africa. In particular, the importance of NTS sepsis is not widely recognized. An important reason is that most hospitals in the region lack adequate microbiological facilities. Epidemiologic data on invasive Salmonellosis in sub-Saharan African countries is important for assisting clinical management and development of preventive strategies. We conducted fever surveillance in a district hospital in rural Tanzania. We estimated the case fraction of invasive salmonellosis among our pediatric admissions, examined associations with common co-morbidities and described its clinical features in comparison with other febrile illnesses. We compared our main findings with those from previous studies among children in sub-Saharan Africa.

## Methods

### Study Site and Population

The study was conducted at Teule Hospital, which is the designated district hospital of Muheza in north-eastern Tanzania. The hospital serves a catchment population of about 277,000 of whom 17% are aged less than five years. Child mortality in the area is 165/1000 [5]. The majority of inhabitants reside in rural settings, mainly practicing subsistence farming and informal trade. The area is highly endemic for *Plasmodium falciparum* malaria with perennial transmission and two seasonal peaks coinciding with the short and long rains [6]. HIV sero-prevalence among antenatal clinic attendees was about 7% in 2007 [7].

The Tanzanian Expanded Programme of Immunization includes the following: Bacille Calmette-Guérin, live oral polio, diphtheria-whole cell pertussis-tetanus-hepatitis B and monovalent measles vaccines for children, as well as supplemental tetanus toxoid vaccine for women of child-bearing age. Tanzania had just started immunization against *Haemophilus influenzae* type b in March 2009 and hopes to introduce pneumococcal vaccine in 2010 [8]. Typhoid vaccine is not routinely administered in the country.

### Fever Surveillance Procedures

Prior to the start and during the course of the study, emergency triage and hospital care guidelines were implemented in the ward [9]. On admission, children aged 2 months to 14 years were screened for eligibility during the study hours from 7am to 7pm, Monday-Sunday. Children with fever of 3 or more days prior to admission or fever of less than 3 days but with at least one severity criteria (respiratory distress, deep breathing, severe pallor with respiratory distress, prostration, capillary refill ≥3 seconds, temperature gradient, systolic blood pressure <70 mm Hg, coma, severe jaundice, history of 2 or more convulsions in last 24 hours, hypoglycemia, neck stiffness, bulging fontanel or desaturation) were recruited into the study. All clinical information was recorded in a standard case record form. Treatment was provided as per national guidelines. Outcome was recorded at discharge or death in a discharge form. Surveillance procedures were supervised by experienced study physicians (GM, IH, JD).

### Point of Care and Laboratory Investigations

We collected 1 to 10 millilitres of blood (depending on body weight) from each eligible child. Immediate bedside testing included those for hemoglobin concentration (Hemocue™, Anglholm, Sweden) and blood glucose level (Accu-check™, Roche Diagnostics GmbH, Germany). We performed two types of rapid diagnostic test (RDT) for *P. falciparum* malaria: HRP-2 based (Paracheck™, Orchid Biomedical, Mumbai, India or Parahit™, Span Diagnostics, Surat, India) and LDH based (OptiMAL-IT, DiaMed AG, Switzerland). From each child, thin and thick blood films were prepared, Giemsa-stained and read by experienced laboratory technicians. At least 100 high power microscopic fields of the thin film were examined to exclude the diagnosis of malaria. Blood for culture was inoculated into a BactALERT™ Pediatric-fan bottle (bioMérieux, Marcy l'Etoile, France) and incubated in the BacT/ALERT 3D automated microbial detection system. Blood cultures were processed according to standard methods. Colonies with biochemical reactions on API20E suggestive of *Salmonellae* were confirmed serologically by slide and tube agglutination testing using specific O and H antisera (Becton Dickinson, NJ, USA). Sera were tested for the presence of HIV-1 and HIV-2 antibodies according to the National HIV rapid testing algorithm [10] using Capillus HIV-1, HIV-2 test (Trinity Biotech, Bray, Ireland) or SD Bioline (Standard Diagnostics, Kyonggi-do, Korea) followed by Determine HIV-1/2 test (Abbott Laboratories, IL, USA) if the first test was positive. Discordant results were resolved by a third antibody test, Unigold (Trinity Biotech, Bray, Ireland), which if positive rendered the sample as positive and if negative, the sample was considered as negative. Children aged less than 18 months with positive results were not tested by polymerase chain reaction for viral antigen and for that reason were excluded in the final analysis.

### Data Management, Definitions and Analysis

Data were double-entered into custom-made data entry programs using MS-Access (Microsoft Corp, VA, USA). Data management programs included error, range and consistency check programs.

Fever was defined as history of a rise in body temperature as recalled by a care-giver or presence of axillary temperature ≥37.5°C on presentation. Bacteremia was defined as fever with isolation of pathogenic bacteria from blood culture, further classified as those caused by *S. typhi* (typhoid fever), NTS, and other (non-*Salmonellae*) pathogenic bacteria. Malaria was defined as fever with a positive RDT or blood film. HIV infection was defined as a positive Capillus test or SD bioline, confirmed either by a positive Determine HIV-1/2 or a positive Unigold test. Low maternal education was considered as schooling to less than the National Curriculum standard 7. Diarrhea was defined as loose or watery stools ≥3 times per day. A seizure was regarded as abnormal movements with altered consciousness. Desaturation was defined as oxygen saturation less than 90% measured by pulse oximetry. Acute severe malnutrition was defined as the presence of bilateral pedal edema or severe wasting. We also assessed the mid-upper arm circumference (MUAC) of children between 12 to 59 months of age. Signs of shock were temperature gradient in the lower extremities and delayed capillary refill of ≥3 seconds or systolic blood pressure <70 mm Hg. Signs of dehydration were delayed skin pinch >2 seconds or sunken eyes. Prostration was defined as inability to sit unsupported (for children over 9 months of age) or to drink/breastfeed. Coma was defined as Blantyre coma score ≤2 for children less than 2 years of age or a Glasgow coma score ≤10 for older children. Hypoglycemia was defined as blood glucose level of <2.5 mmol/litre. Anemia was defined as hemoglobin of <8 g/dl and severe anemia <5 g/dl.

To assess potentially important distinguishing factors, we classified the cases into 5 non-mutually-exclusive groups: typhoid fever, invasive NTS infection, other pathogenic bacteremia, malaria, and those without bacteremia and malaria. Comparisons of categorical data were made using the Chi square or Fishers' Exact test, as appropriate. Comparisons of continuous data were made using student's t-test for data with equal variance or Welch's t-test for those with unequal variance. All analyses were performed using Stata™ v 10.0 (Stata Corp., Tx, USA).

### Literature Review

We conducted a literature review to compare our main findings with those from previous studies. Potential articles for inclusion were identified by direct searches of the MEDLINE database through PubMed. We included facility-based studies of children ≤16 years in sub-Saharan Africa that reported case fractions of *S. typhi* and NTS infection from sterile-site specimen (blood, CSF, lung or joint aspirate) bacterial cultures. The searches were restricted to publications from 1987 to date. For study sites with several publications generated from the same study population, only one citation was included unless the time period varied. We also conducted supplementary searches of the references in retrieved articles. Abstracts were reviewed and if relevant, the article was included.

### Ethics

The fever surveillance was conducted following the principles governing biomedical research involving human subjects. Prior written informed consent was obtained from the parent or guardian of each eligible child. Pre-test counseling was provided before HIV testing in accordance with local guidelines. The study was approved by the National Institute for Medical Research, Tanzania (NIMR/HQ/R.8a/Vol.IX/666) and the International Vaccine Institute - Institutional Review Boards, South Korea (IRB# 2007-017).

## Results

**Figure 1:**
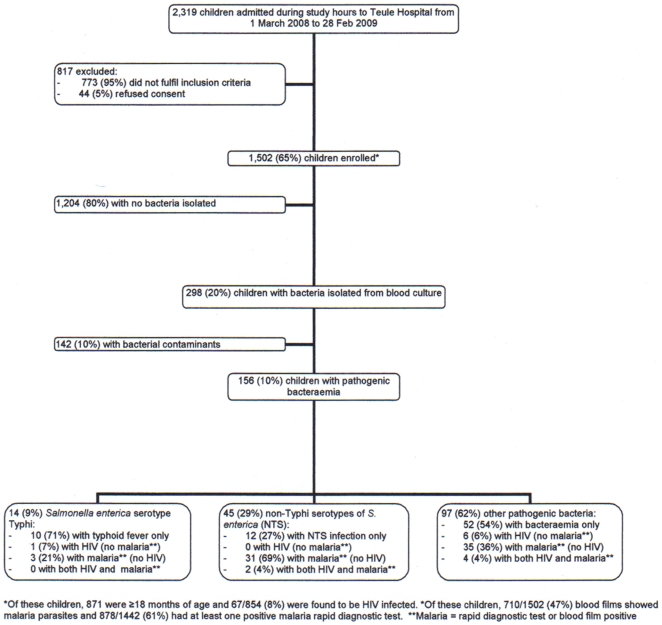
The study population.

From 1 March 2008 to 28 February 2009, 2319 children were admitted to Teule Hospital during study hours (Figure 1). After excluding 817 (35%) children who did not fulfill inclusion criteria or whose parents declined to participate in the study, 1,502 (65%) children were enrolled. Bacteria were isolated from the blood of 298 (20%) of these children, of which 142 (10%) were considered as likely contaminants. Each of the 156 children with pathogenic bacteremia had only a single organism isolated from their blood culture. Of the 156 (10%) bacteremic children, 14 (9%) had *S. typhi*, 45 (29%) had NTS and 97 (62%) had other pathogenic bacteria. Thus, invasive salmonellosis accounted for 59/156 (38%) bacteremic children.

We compared our findings with those from previous studies among children in sub-Saharan Africa (Table 1). Sixteen articles from 8 countries fulfilled our selection criteria [11]–[26]. In this series, the isolation rates of pathogenic bacteria ranged from 1 to 46%, the case fraction of *S. typhi* ranged from 0 to 42% and of NTS from 9 to 84%, depending on the study sampling frame. The number of NTS isolates for every one *S. typhi* ranged from 0.8 to 166.

**Table 1:** Facility-based studies of invasive salmonellosis in children in sub-Saharan Africa (arranged by study site and chronologic order).

Country (urban or rural population)	Author, year (Reference number)	Study time frame	Age group	Sampling criteria	Number (specimen type)	Pathogenic bacteria (%)	*S typhi* (% of pathogenic bacteria)	Non-typhoidal salmonellae (% of pathogenic bacteria)	Number of NTS isolates for every 1 *S typhi*
Tanzania (rural)	This paper	2008–2009	2 months to 14 years	Admitted with ≥3 days fever or < 3days fever but with severity criteria	1502 children (blood)	156 (10)	14 (9)	45 (29)	3
1. Gambia (rural)	Enwere et al. 2006 (11)	2003–2004	2–29 months	In and outpatients with signs of infection and a temperature of ≥38°C. Carriedout as part of a pneumococcal vaccine trial	7369 specimens (blood, CSF, lung aspirate)	330 (4)	0 (0)	92 (28)	No *S typhi*
2. Gambia (rural)	O'Dempsey et al. 1994 (12)	1989–1991	<5 years	Admitted with pneumonia, meningitis or suspected septicaemia	1162 children (blood)	184 (16)	11 (6)	19 (10)	2
3. Gambia (urban)	Mabey et al. 1987 (13)	1979–1984	children	Admitted	(blood)	247	45 (18)	71 (29)	2
4. Kenya (rural)	Williams et al. 2009 (14)	1998–2008	<14 years	All admitted except those with elective procedures or accidents	38441 children (blood)	2157 (6)	9 (0.4)	211 (10)	23
5. Kenya (rural)	Brent et al. 2006 (15)	2003	<5 years	Randomly selected 10% of outpatients, excluding those admitted to hospital within the previous 10 days	1093 children (blood)	22 (2)	0 (0)	2 (9)	No *S typhi*
6. Kenya (rural)	Berkley et al. 2005 (16)	1998–2002	<13 years	All admitted except those with elective procedures or accidents	19339 children (blood)	228 (13)	1 (0.4)	166 (73)	166
7. Kenya (rural)	Berkley et al. 1999 (17)	1993–1996	children	Admitted with severe malaria	783 children (blood)	42 (5)	0 (0)	6 (14)	No *S typhi*
8. Malawi (mixed)	Bronzan et al. 2007 (18)	1996–2005	≥6 months–≤15 years	Admitted with severe malaria	1388 children (blood)	64 (5)	1 (2)	37 (58)	37
9. Malawi (mixed)	Walsh et al. 2000 (19)	1996–1997	≤15 years	Admitted with suspected bacteremia (febrile or very ill without an adequate explanation by physical examination or blood film) or who remained febrile after treatment for malaria	2123 children (blood)	365 (17)	15 (4)	140 (38)	9
10. Mozambique (rural)	Sigauque et al. 2009 (20)	2001–2006	<15 years	All admitted <2 years of age. Those 2–14 years with temperature ≥39°C or with severity criteria	19 896 children (blood)	1550 (8)	3 (0.2)	401 (26)	134
11. Nigeria (urban)	Falade et al. 2009 (21)	2005–2006	2–59 months	Admitted with features of community-acquired pneumococcal disease	330 children (blood)	95 (29)	0 (0)	15 (16)	No *S typhi*
12. Rwanda (mixed)	Lepage et al. 1987 (22)	1984–1985	<15-years	Outpatients with temp ≥39°C excluding those admitted to hospital within the preceding 3 months and those with measles up to 10 days after onset of rash	14032 children (blood)	112 (1)	47 (42)	36 (32)	0.8
13. Uganda (urban)	Bachou et al. 2006 (23)	2003–2004	<5 years	Admitted with severe malnutrition	445 children (blood)	76 (17)	5 (7)	28 (37)	6
14. Zaire (rural)	Bahwere et al. 2001 (24)	1989–1990	All children	On admission (whether febrile or not)	779 children (blood)	124 (16)	2 (2)	53 (43)	27
15. Zaire (rural)	Cheesbrough et al. 1997 (25)	1990–1992	1–16 years	In and outpatients, fitted into a preset clinical case definition of salmonella bacteraemia	120 children (blood)	55 (46)	11 (20)	35 (63)	3
16. Zaire (rural)	Green et al. 1993 (26)		≤5 years	Admitted with clinically suspected salmonella infection (i.e. persistent fever, no response to anti-malarial treatment)	--- (blood, CSF, joint aspirate)	206	34 (17)	172 (84)	5

Of the 1,502 children enrolled, 806 (54%) were >2 months to 2 years, 520 (34%) were >2 years to 5 years and 176 (12%) were >5 years of age (Table 2). We ranked the bacterial pathogenic isolates according to frequency. Overall and among children less than 5 years of age, NTS was the principal organism. *S. typhi* was the most common isolate among those over 5 to 14 years of age. Other commonly isolated bacterial pathogens included *Escherichia coli* (27/156 or 17%) and *Haemophilus influenzae* type B (20/156 or 13%). There were 8 (5%) *Streptococcus pneumoniae* isolates.

**Table 2:** Bacterial species isolated from 156 children with bacteremia, ranked* according to frequency.

	>2 m to 2 y (n = 806)	Rank	>2 y to 5 y (n = 520)	Rank	>5 y (n = 176)	Rank	Total (%) (n = 1,502)	Overall rank
**Gram-positive**								
*-Streptococcus pneumoniae*	4	5	4	4	0	8	**8 (5.1)**	**8**
-beta haemolytic Streptococci, Group A & C	5	4	2	9	2	6	**9 (5.8)**	**7**
*-Staphylococcus aureus*	2	9	0	10	3	2	**5 (3.2)**	**10**
**Gram-negative**								
*-Salmonella typhi*	1	10	4	4	9	1	**14 (9.0)**	**4**
-Nontyphoidal salmonella species	30	1	12	1	3	2	**45 (28.8)**	**1**
-*Haemophilus influenzae* (type B)	17	3	3	7	0	8	**20 (12.8)**	**3**
*-Escherichia coli*	21	2	5	2	1	7	**27 (17.3)**	**2**
-Acinetobacter species	3	7	3	7	0	8	**6 (3.8)**	**9**
-Non-fermenters	4	5	4	4	3	2	**11 (7.1)**	**5**
Other**	3	7	5	2	3	2	**11 (7.1)**	**5**
**All pathogenic bacteria**	**90**		**42**		**24**		**156 (100)**	
**Contaminants** ***	**94**		**38**		**10**		**142**	
**Total**	**184**		**80**		**34**		**298**	

*Rank (by age group and overall) was the same for organisms with the same frequency.

**Species included: Candida (n = 1), Citrobacter braakii (n = 1), Haemophilus parainfluenzae (n = 2), Pantoea species (n = 1), Gram negative rods not identified (n = 6).

***Species included: Bacillus (n = 19), Diphtheroids (n = 6), Micrococcus (n = 6), alpha-hemolytic Streptococcus viridans (n = 3), coagulase negative Staphylococcus (n = 98), yeast (n = 5), mixed bacterial species (n = 4), Gram positive rods not identified (n = 1).

We present the percentage of malaria and HIV co-infections among children with *S. typhi*, NTS, and other pathogenic bacteria (Figure). Considering those with a positive RDT or blood film as having malaria, children with invasive NTS infection were more likely to also have malaria (33/45 or 73%) compared to those with typhoid fever (3/14 or 21%, p value <0.01) and other bacteremia (39/97 or 40%, p value <0.01). The proportion of those with a positive RDT and negative blood film was highest among those with invasive NTS infection (20/45 or 44%) compared to those with typhoid fever (2/14 or 14%, p value >0.05) and other bacteremia (13/97 or 13%, p value <0.01).

We compared the admission clinical features and outcome of typhoid fever, invasive NTS infection, other pathogenic bacteremia, and malaria cases with those of children without bacteremia and malaria (Table 3). Age was an important distinguishing characteristic. Children with typhoid fever had the highest mean age (8 years) and were significantly older than children without bacteremia and malaria (p value <0.01). Typhoid fever patients had the longest duration of fever prior to admission (mean  = 10 days, p value  = 0.02). Bacteremia (except for those due to *S*. *typhi*) and malaria were significantly associated with low maternal education. Malaria patients were less likely to present with cough and diarrhea (p value <0.01) compared to children without bacteremia and malaria. Children with other pathogenic bacteraemia and malaria were more likely to have seizures and present in coma (p value <0.01) compared to children without bacteremia and malaria. Compared to the other groups, children with invasive NTS infection had most frequently received an antimalarial medication prior to admission (35/45; 78%). Although uncommon, children with invasive NTS were also more likely to be jaundiced (3/45; 7%; p value  = 0.01). Severe palmar pallor, anemia and hypoglycemia were common among invasive NTS infection, other pathogenic bacteremia, and malaria cases but those with invasive NTS had the lowest mean hemoglobin level (5.7 g/dl) and were most frequently hypoglycemic (6/45; 21%). Children with invasive NTS died more frequently (11/45; 24%; p value <0.01) compared to all other groups.

**Table 3:** Clinical features on admission and outcome of febrile cases, by non-mutually exclusive laboratory-confirmed groups*.

	All	Group 1: Typhoid fever (n = 14)	Group 2: Invasive NTS infection (n = 45)	Group 3: Other pathogenic bacteraemia (n = 97)	Group 4: Malaria (n = 947)	Group 5: No bacteraemia and malaria (n = 474)	P value (1vs5)	P value (2vs5)	P value (3vs5)	P value (4vs5)
**Mean age in years; n = 1,502**	2.6	7.5	2.0	2.3	2.7	2.1	0.00	0.64	0.60	0.00
**N (%) male; n = 1,502**	813 (54.1)	3 (21.4)	24 (53.3)	45 (46.4)	501 (52.9)	274 (57.8)	0.01	0.64	0.04	0.09
**N (%) with low maternal education; n = 1,348**	457 (33.9)	3 (30.0)	17 (43.6)	36 (41.4)	314 (37.1)	118 (27.2)	1.00	0.04	0.01	0.00
**Mean days of fever; n = 1,499**	5.0	10.1	6.6	5.3	4.4	5.7	0.02	0.45	0.47	0.00
**N (%) with cough; n = 1,502**	876 (58.3)	8 (57.1)	33 (73.3)	66 (68.0)	490 (51.7)	333 (70.3)	0.37	0.74	0.72	0.00
**N (%) with diarrhea; n = 1,493**	287 (19.2)	6 (42.9)	10 (22.2)	18 (18.8)	129 (13.7)	137 (29.0)	0.37	0.39	0.04	0.00
**N (%) with vomiting; n = 1,491**	737 (49.4)	10 (71.4)	24 (53.3)	41 (42.7)	466 (49.6)	234 (49.7)	0.17	0.76	0.22	1.00
**N (%) with seizures; n = 1,495**	220 (14.7)	0 (0)	2 (4.4)	15 (15.5)	180 (19.1)	34 (7.2)	0.61	0.76	0.02	0.00
**N (%) with coma; n = 1,476**	94 (6.4)	0 (0)	2 (4.7)	12 (12.4)	77 (8.3)	10 (2.1)	1.00	0.27	0.00	0.00
**N (%) received antimalarial; n = 1,485**	921 (62.0)	8 (61.5)	35 (77.8)	64 (66.7)	585 (62.2)	287 (61.6)	1.00	0.04	0.42	0.82
**N (%) received antimicrobial; n = 1,485**	247 (16.7)	2 (14.3)	9 (20.0)	24 (24.7)	104 (11.2)	121 (25.9)	0.53	0.48	0.90	0.00
**Mean axillary temp on admission in °C; n = 1,483**	38.1	38.5	38.1	38.2	38.1	38.0	0.07	0.68	0.15	0.10
**Mean heart rate on admission; n = 1,465**	155.0	122.6	163.0	153.6	156.6	153.0	0.00	0.02	0.86	0.02
**N (%) with desaturation; n = 1,475**	85 (5.8)	1 (7.7)	3 (6.8)	5 (5.2)	43 (4.6)	37 (8.0)	1.00	1.00	0.52	0.01
**N (%) with severe palmar pallor; n = 1,498**	434 (29.0)	0 (0)	28 (62.2)	33 (34.0)	383 (40.5)	34 (7.2)	0.61	0.00	0.00	0.00
**Among children 12–59 months, n (%) with MUAC<12.5 cm; n = 859**	47 (5.5)	0 (0)	3 (12.5)	2 (4.2)	16 (2.6)	28 (13.3)	1.00	1.00	0.08	0.00
**N (%) with sign of severe malnutrition; n = 1,499**	47 (3.1)	0 (0)	2 (4.5)	5 (5.2)	14 (1.5)	27 (5.7)	1.00	1.00	1.00	0.00
**N (%) with sign of shock; n = 1,495**	55 (3.7)	0 (0)	2 (4.5)	7 (7.3)	35 (3.7)	13 (2.8)	1.00	0.37	0.06	0.44
**N (%) with sign of dehydration; n = 1,459**	60 (4.1)	0 (0)	4 (9.1)	5 (5.4)	18 (1.9)	36 (7.9)	0.61	0.77	0.52	0.00
**N (%) with jaundice; n = 1,475**	18 (1.2)	0 (0)	3 (6.8)	1 (1.1)	14 (1.5)	3 (0.7)	1.00	0.01	0.52	0.21
**N (%) prostrated; n = 1,292**	337 (26.1)	2 (20.0)	7 (17.5)	32 (37.6)	269 (32.4)	52 (13.0)	0.63	0.46	0.00	0.00
**N (%) with neck stiffness or bulging fontanelle; n = 1,491**	24 (1.6)	0 (0)	1 (2.2)	5 (5.2)	6 (0.6)	13 (2.8)	1.00	1.00	0.21	0.00
**N (%) with impaired consciousness; n = 1,481**	68 (4.6)	1 (7.1)	3 (6.8)	8 (8.3)	57 (6.1)	7 (1.5)	0.21	0.05	0.00	0.00
**Mean blood glucose in mmol/litre; n = 840**	5.4	3.8	4.5	4.8	5.3	5.5	0.06	0.01	0.01	0.20
**N (%) with hypoglycemia; n = 840**	63 (7.5)	1 (25.0)	6 (21.4)	7 (12.3)	48 (7.8)	8 (4.3)	0.18	0.00	0.05	0.14
**Mean hemoglobin in g/dl; n = 1,478**	8.0	10.2	5.7	7.9	7.1	9.7	0.47	0.00	0.00	0.00
**N (%) with anemia; n = 1,478**	674 (45.6)	2 (14.3)	37 (82.2)	45 (46.9)	550 (59.3)	95 (20.2)	0.75	0.00	0.00	0.00
**N (%) with severe anemia; n = 1,478**	274 (18.5)	0 (0)	18 (40.0)	15 (15.6)	244 (26.3)	23 (4.9)	1.00	0.00	0.00	0.00
**Among children ≥18 months, n (%) HIV infected; n = 854**	67 (7.8)	1 (8.3)	2 (9.1)	10 (20.8)	36 (5.7)	24 (13.3)	1.00	0.75	0.25	0.00
**Died; n = 1,502**	92 (6.1)	1 (7.1)	11 (24.4)	11 (11.3)	54 (5.7)	27 (5.7)	0.57	0.00	0.07	1.00

*Comparisons of categorical data were made using the Chi square or Fishers' Exact test, as appropriate. Comparisons of continuous data were made using student's t-test for data with equal variance or Welch's t-test for those with unequal variance.

## Discussion

In our study population of children between 2 months to 14 years of age, *Salmonellae* ranked as the most common cause of bacteraemia. Our over-all isolation rate of pathogenic bacteria was similar to that in previous studies that used similar sampling criteria [16], [20]. But reviewing previous reports, we noted considerable variation in the case fraction of *S. typhi* and NTS infections [11]–[26]. Other important lessons were gleaned from our review of the literature. First, we found that data was available only from a few sub-Saharan African countries with the majority of studies having been conducted in research centres in the Gambia, Kenya, Malawi, and Mozambique. Second, in all but one report [22], NTS outnumbered *S. typhi* infections by several-fold. In many sites, pediatric typhoid fever was not detected at all. This is in marked contrast to findings from Asia where a high burden of disease is seen even in the youngest age groups [27], [28]. Third, although NTS was isolated in all studies, there was a wide range in the case fractions by geographic location and time period. For example, in Kilifi, the case fraction of NTS decreased from 73% (1998 to 2002) to 10% (1998–2008) [14], [16]. Interestingly, the burden of malaria in the area decreased substantially during this time period. It was estimated that hospital admissions for malaria decreased from 18·4 per 1000 children in 2003 to 3·4 in 2007 [29].

Malaria has long been suspected to increase the risk and contribute to the seasonality of invasive non-typhi salmonellosis [3]. The common occurrence of severe NTS septicemia during malaria outbreaks was first reported in British Guiana in the 1920's [30]. Duggan and Beyer suggested an association between invasive salmonellosis and malaria in Nigerian children [31]. In 1987, Mabey et al found that young Gambian children with NTS septicemia were more anemic and more likely to have evidence of recent malaria than were children of the same age with other forms of septicaemia [13]. In Malawi, studies have showed an association between NTS bacteraemia and severe anaemia [4], [18], [32]. In Kenya, Brent et al subsequently found that three-fourths of NTS patients with anaemia had evidence of either current or recent malaria [33]. In this study, NTS infections were significantly associated with prior antimalarial treatment and malarial complications (severe anemia, jaundice and hypoglycemia). And similar to a previous report [33], we found that a positive RDT with a negative blood smear for malaria was most common among the NTS group, suggesting a past rather than a current malaria infection. This supports the long-held hypothesis [13] that malaria is the preceding event which predisposes these children to invasive NTS infection. The mechanism underlying the association between malaria and NTS is incompletely understood. It is possible that the metabolic, haemodynamic or inflammatory processes that can occur during severe malaria also predispose to invasive bacterial disease [3]. Malnutrition was associated with NTS bacteremia among children in Kenya [33] but we could not confirm this in our study. We were also unable to explore other risk factors that have been associated with NTS infections in Africa such as contaminated food and water, animal contact, sickle cell disease, schistosomiasis, and recent antimicrobial use [3].

In this study, typhoid fever cases were more common in older children and presented with a longer duration of fever. In contrast, the non-typhoid bacteremia cases, as well as malaria, tended to occur in young children, particularly of poorly-educated women. It is likely that low educational attainment is a marker for low socio-economic status. NTS infection was associated with a considerable increased risk for death.

Our study has several limitations. First, it is well known that blood cultures are insensitive for detecting bacteremia. Small blood volumes for culture especially from the younger and sicker patients, as well as the prior use of antibiotics, further decrease the sensitivity of blood cultures. Among our participants, 17% admitted to antibiotic use prior to admission. Thus, it is likely that we have underestimated the case-fractions of hospitalized invasive salmonellosis. This may also have led to an underestimate of the overall number of bacteremic children and contamination of the comparison groups due to false-negative diagnoses. Second, we compared and contrasted features of non-mutually-exclusive groups: typhoid fever, invasive NTS infection, other pathogenic bacteremia, malaria, and those without bacteremia and malaria. It was necessary to use these non-mutually exclusive groups as it is not possible to determine whether malaria or bacteremia was the precipitating event that led to admission. Factors associated with each group could modify the severity of the illness and be responsible for the differences in clinical picture. On the other hand, this comparison yielded characteristics that clinicians could potentially use to help distinguish invasive Salmonellosis cases from other hospitalized patients. Third, we were not able to include children less than 2 months of age in the current fever surveillance since these children were admitted and cared for in a separate area of the hospital. Fourth, serotyping of the NTS isolates was beyond the scope of the study but would have added interesting information. Although NTS cases are a rather heterogeneous group of diseases, previous reports have shown that *S. typhimurium* and *S. enteritidis* are the predominant serotypes among African children [3], [18]–[20], [32]–[34].

In summary, we found that in a malaria endemic region in Tanzania, invasive salmonellosis is an important cause of hospitalized febrile diseases among children. Invasive NTS disease is associated with a high risk for death. Certain suggestive clinical features (such as older age and long duration of fever for typhoid whereas concomitant malaria, anemia, jaundice and hypoglycemia for NTS infection) may be used to distinguish invasive salmonellosis from other severe febrile illness on presentation. We shall continue our fever surveillance in Teule hospital to follow trends in the occurrence and clinical picture of these infections in our community.
